# AGE AND LOSARTAN EFFECTS ON MITOCHONDRIAL PROTEOME IN MICE

**DOI:** 10.1093/geroni/igae098.3633

**Published:** 2024-12-31

**Authors:** Adam Said, Michael Bene, Michael Abadir, Simion Kreimer, Ruth Marx, Laura Powell, Jeremy Walston

**Affiliations:** Emory College of Arts and Sciences, Atlanta, Georgia, United States; Johns Hopkins School of Medicine, Baltimore, Maryland, United States; University of Maryland, Woodstock, Maryland, United States; The Mass Spectrometry and Proteomics Facility Johns Hopkins University School of Medicine, Baltimore, Maryland, United States; Johns Hopkins University, Baltimore, Maryland, United States; Johns Hopkins University, Baltimore, Maryland, United States; Johns Hopkins University, Baltimore, Maryland, United States

## Abstract

Loss of mitochondrial function with age is associated with reduced organ functional performance and predispose individuals to age-related diseases. Current studies suggest the renin-angiotensin system significantly interacts with mitochondrial function. This study investigated the effects of aging and Losartan, an angiotensin II type 1 receptor blocker, on the mitochondrial proteome in mice. Mitochondrial samples from young mice (3 months), aged mice (24 months), and aged mice treated with Losartan (24 and 33 months) were analyzed using Cytoscape. Aged (24 months) mice had a marked downregulation of beta-oxidation and amino acid degradation proteins compared to young (3 months) mice. In contrast, chaperone and muscle contraction proteins were upregulated in older mice. Additionally, the TCA cycle proteins showed significant changes with age, with some proteins becoming increased in abundance while others became significantly less abundant. Overall, there was not a clear regulation pattern, suggesting a further complexity in mitochondrial energy production with age. Losartan treatment in 24-month-old mice upregulated the TCA cycle and beta-oxidation proteins. In contrast, chaperone and muscle contraction proteins were downregulated. This trend opposes the aging effect and suggests the regulatory effects of Losartan. At 33 months, there were no significant changes in any protein groups of losartan-treated mice. This difference in expression suggests that more advanced age may prevent the regulatory effects of losartan. In line with previous studies, these results suggest that Losartan has the potential to compensate for age-related functional decline. Losartan is a potential pharmacological intervention that may reverse aging-related mitochondrial proteome decline.

